# Performance Evaluation of an Object Detection Model Using Drone Imagery in Urban Areas for Semi-Automatic Artificial Intelligence Dataset Construction

**DOI:** 10.3390/s24196347

**Published:** 2024-09-30

**Authors:** Phillip Kim, Junhee Youn

**Affiliations:** Department of Future & Smart Construction Research, Korea Institute of Civil and Building Technology, Goyang-si 10223, Republic of Korea; ph.doit@gmail.com

**Keywords:** semi-automatic object labeling, dynamic spatial information, drone

## Abstract

Modern image processing technologies, such as deep learning techniques, are increasingly used to detect changes in various image media (e.g., CCTV and satellite) and understand their social and scientific significance. Drone-based traffic monitoring involves the detection and classification of moving objects within a city using deep learning-based models, which requires extensive training data. Therefore, the creation of training data consumes a significant portion of the resources required to develop these models, which is a major obstacle in artificial intelligence (AI)-based urban environment management. In this study, a performance evaluation method for semi-moving object detection is proposed using an existing AI-based object detection model, which is used to construct AI training datasets. The tasks to refine the results of AI-model-based object detection are analyzed, and an efficient evaluation method is proposed for the semi-automatic construction of AI training data. Different F_Beta_ scores are tested as metrics for performance evaluation, and it is found that the F_2_ score could improve the completeness of the dataset with 26.5% less effort compared to the F_0.5_ score and 7.1% less effort compared to the F1 score. Resource requirements for future AI model development can be reduced, enabling the efficient creation of AI training data.

## 1. Introduction

Recent improvements in deep learning algorithms and computer specifications, alongside the availability of diverse datasets, have driven research on capturing different types of information by applying computer vision techniques to image data, which has found applications in various fields. Such research is being conducted in industrial fields and also in the public sector to solve social problems, and the range of images used is continuously broadening. Although CCTV and aerial imagery have primarily been used in the public sector, the developments in drone operation and manufacturing technology have expanded the use of computer vision techniques to drone imagery. Drone-acquired images cover a smaller area than that of images acquired in conventional aerial imagery but can capture relatively high-resolution images. The lower operating costs enable more frequent image acquisition, making them highly useful in practice. Key research examples of public sector applications using drone imagery and object detection techniques include natural environment monitoring, disaster situation assessment, and urban environment management.

A common example of the application of drone imagery to the natural environment is image-based wildlife detection. Hong et al. recognized birds from images captured in various situations [1]. Shao et al. proposed a system for detecting and counting cattle based on drone-captured images for the management of free-range livestock [2]. Wittstruck et al. used drone imagery to estimate the yield, weight, and other factors of pumpkins before harvest to commercialize pumpkin farming [3]. Chen et al. proposed a system for estimating the number of strawberry flowers and fruits to predict yield more efficiently and accurately, providing a basis for creating future yield distribution maps [4]. Yuan and Choi detected apple buds according to their stage of development to determine the heat requirements of orchards. In addition to assessing the condition of wildlife, research has also been conducted to understand natural phenomena [5]. Gao et al. developed a system to monitor air quality indices based on 360° aerial panoramic images captured by a drone [6]. Lyu et al. used the volume of green areas captured by drones as foundational data for high-resolution PM2.5 simulations [7]. Mukudan et al. derived a model that classified the air quality indices into three categories by applying hyperspectral image processing techniques in combination with deep learning to drone-captured images [8]. In addition, satellite imagery, although not captured by drones, has been used to analyze fog, sea mist, and other phenomena [9,10].

In the field of disaster prediction and response, drone imagery is often used to detect structures, earthquakes, and wildfires. Pi et al. constructed a dataset called Volan 2018 as a basis for disaster impact and damage information, and the authors used images collected from drones and helicopters of the damage caused by hurricanes in the United States between 2017 and 2018 [11]. Kyrkou et al. proposed an image analysis algorithm called EmergencyNet that uses drones to respond to disasters [12]. Lygouras et al. integrated drone images with the global navigation satellite system to detect and locate people affected by a disaster [13]. In addition, drone imagery has been actively used in the field of wildfires for wildfire detection, [14,15] monitoring [16,17], the prediction of potential wildfires [18], and optimization of movement trajectories based on network coverage for wildfire detection with drone imagery [19]. In addition, research has been conducted using drone imagery to automatically extract information on building damage after an earthquake [20,21] and review structural displacements of buildings [22,23].

Finally, in the field of urban environment detection, the detection of moving objects, such as vehicles, people, and particulate matter, as well as abnormal behavior, has been investigated. The field where drone imagery has been most widely used could most likely be traffic monitoring, including traffic volume assessment. Early drone-based traffic surveillance was primarily concerned with detecting vehicles from video footage to measure traffic volume and speed or to track individual objects [24,25]. As object detection and tracking technologies have advanced, their applications for traffic monitoring and management have expanded accordingly. Wang et al. proposed a system for comprehensive traffic diagnosis [26], in which images of roads and intersections are captured with drones on site, sends them to an edge computing server, and uses a deep learning model to identify and track vehicles. The system also monitors traffic violations, such as parking on shoulders and speeding, and uses the results to analyze traffic volumes and vehicle speeds. Guzman and Baeza proposed a system for urban traffic management that combines the use of drones with traffic signals [27]. By identifying traffic congestion areas using drones and relaying this information to ground traffic signals, the ground traffic flow can be controlled, creating an efficient, sustainable, and flexible urban transportation system that can contribute to the development of refined and responsive smart city traffic management solutions. The application of drones for urban traffic management has evolved from simple monitoring tasks, such as counting vehicles and measuring speed on specific roads, to building systems for managing urban traffic environments. The active use of drones will enable the monitoring of the traffic environment from a different perspective than conventional CCTV-based urban traffic monitoring. However, traffic environment monitoring using drones requires the collection of information regarding various moving objects (such as people and vehicles) moving in the city through regular and periodic drone operations.

Deep learning object detection techniques are widely used to detect objects in drone images, and various metrics are used to evaluate their performance. Zhou et al. (2022) developed a model for small object detection based on drone imagery by modifying the backbone of the YOLO v4 model and using data augmentation techniques, such as rotation and cropping, with mean average precision (mAP), mAP50, and detection time as the evaluation metrics [28]. Sun et al. compared the performance of object detection in drone mages with a single-hot detector and faster region-based convolutional neural networks, using metrics such as computation time, mAP, and memory usage [29]. Studies on the development of lightweight and efficient object detectors based on edge computing technology for deployment on drones [30,31] and the development of drone-based object detectors for intelligent traffic management [32,33] have also used mAP as an important evaluation metric. mAP50, mAP, etc., are considered representative metrics for evaluating the performance of object detectors. The mAP is determined by calculating the AP through a comprehensive evaluation of precision and recall and then averaging the calculated values across all classes. Compared to using precision alone, evaluating model performance through a complex process effectively reflects the capabilities of complex object detection models and enables assessment across different thresholds. However, due to its computational complexity, mAP suffers from long computation time on large datasets and does not reflect performance differences between classes. Moreover, it focuses on comprehensive performance evaluation based on the results of object detection by the model. Consequently, it is unsuitable for evaluating the performance of modifying object detection results to use as training data for the AI model.

The development of deep learning-based object detection techniques requires the manual labeling of target objects and the creation of training data, which is associated with a considerable amount of time and cost. Therefore, automatic or semi-automatic generation of training data for AI model training can reduce the time required to generate the basic data for AI model development. In this study, a method for evaluating the detection performance that uses an existing deep learning model for the semi-automatic generation of AI training data was proposed. Prior research on facilitating the creation of datasets has primarily focused on developing new models. Approaches have included applying labels without bounding boxes [34], providing labels for only a subset of the data [35], or using human assistance for partial labeling [36], thereby opting for methods that improve the labeling process. However, the use of conventional commercial labeling methods is rare, and this study distinguishes itself by using conventional labeling techniques. Instead of focusing on the evaluation of object detection performance, e.g., by using the conventional mAP, the proposed method evaluates the performance based on the usability of the detection results. Thus, the objective of this study is to facilitate the efficient generation of AI training data by deriving the evaluation criteria for efficiently refining the detected data using existing models. The detection of moving objects from drone images captured at different altitudes in urban areas is evaluated using the F-beta score, derived from a combination of recall and precision, and a method for determining the thresholds to create the AI training data was formulated.

## 2. Materials and Methods

### 2.1. Selection of Object Labeling Methods

The rapid progress in AI technology has led to various tasks that use AI. The development of AI models for problem-solving fundamentally requires large volumes of data. Moreover, it is necessary to collect raw data and label the ground truth for AI training. According to a study by the National Information Society Agency of Korea, approximately 75% of the costs incurred by AI/data companies in Korea during the data creation process are related to data collection, refinement, and labeling [37]. The Korean Copyright Commission reports that 80% of AI projects involve tasks related to the collection, refinement, and labeling of the data used in the development of AI models [38]. A significant portion of the workforce and resources allocated to AI model development is dedicated to data collection, refinement, and processing, resulting in bottlenecks in the workflow. The process of creating AI training data is no longer labor-intensive but is automated by incorporating existing models and technologies, and existing bottlenecks could be eliminated by reducing the cost and duration of the model development process.

The process of creating training data to detect objects in image data, such as the drone images used in this study, can be broken down into individual steps: selection of the target objects in the images, their identification based on the classification system, and their annotation. Annotation refers to the process of marking notes on the source data, which can take various forms based on the functional purpose of the AI model. Based on the type of image data and functional purpose, annotation methods can be categorized. For static images, the task can be categorized into classification, object detection, segmentation, and object recognition. The applicable annotation methods for static images are class labels, bounding boxes, polylines, polygons, and key points. In the case of a dynamic image, the task could be divided into classification, object detection, and tracking. The applicable annotation methods are class label, bounding box, polyline, cuboid, and key points [39,40].

The objective of this study is to detect moving objects on roads to collect dynamic spatial information using static images. Applicable methods include bounding boxes, polygons, and polylines. The annotation methods have different characteristics [41,42]. Bounding box is the most commonly used annotation method for object detection tasks in images. The objects are enclosed by a rectangular box, and the classes are distinguished using this annotation method. This ensures the entire object is included while minimizing the empty space within the box. Although this is the easiest method, it has limitations in displaying the object shapes. Polygon is the tracing of the outline of the visible area of the object with points in a polygonal shape. This can be used to correct addressing errors caused by empty spaces outside the object. More detailed annotations can provide better data but require more labor. Polyline uses lines with multiple points to annotate specific areas, primarily to distinguish sidewalks, lanes, and so forth. It is especially used when the shape has special characteristics.

The bounding box, which is commonly used as an annotation method for object detection in datasets of drone imagery, such as VisDrone, UAVDT, and AI-TOD, was selected as the annotation method in this study [43,44,45].

### 2.2. Data Collection and Processing

Drone footage was collected from Ilsanseo-gu in Goyang-si and Seodaemun-gu in Seoul, South Korea. The specifications of the drones and filming schedule used for collecting the footage are listed in Table 1.

The objective of this study is to detect objects using object detection models and use the results as AI training data with minimal modifications. To detect objects using the model and evaluate the performance, it is necessary to establish the ground truth by annotating the captured footage. Therefore, the ground truth was created by a manual labeling process, and the types of moving objects for labeling were classified into five categories, which are listed in Table 2.

Different altitudes were used during the filming to ensure diversity in the footage. The resolution, number of videos, and number of objects obtained through manual labeling per video are summarized in Table 3.

### 2.3. Selection of the Object Detection Algorithm

Deep learning algorithms for object detection are categorized into one-stage and two-stage detectors based on the detection method, which differ in detection time and accuracy. Since this study aims to use existing deep learning algorithms to build AI training data, one-stage detectors with shorter detection times are considered despite their relatively lower accuracy. Five criteria are established for the selection of the algorithm to be used in this study, as listed in Table 4.

Upon reviewing various algorithms against the established criteria, two algorithms were selected. The comparison between the two selected algorithms based on the set criteria is presented in Table 5.

**Figure 1:**
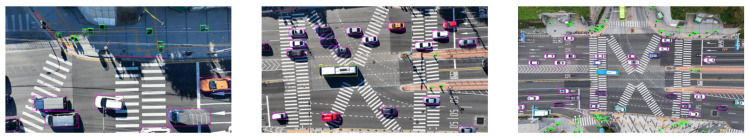
Example of object detection using the PP-YOLOE+ algorithm.

The PP-YOLOE+ algorithm is an optimized model of its predecessor PP-YOLOv2 using a powerful anchor-free backbone and a neck consisting of CSPRepResStage, PAN (path aggregation network), ET-head (efficient task-aligned head), and TAL (task alignment learning). The model was pretrained using the Object365 dataset [46]. Figure 1 shows examples of object detection by applying the PP-YOLOE+ algorithm to drone footage captured at different altitudes (25 m, 50 m, 75 m).

PP-YOLOE+SOD (small object detection) is an improved model of PP-YOLOE specifically designed for small object detection. It uses a vector-based dynamic fuzzy logic algorithm related to the distribution of the dataset and a central prior optimization strategy [47]. Additionally, the model includes a transformer module added to the neck (feature pyramid network), along with an added P2 layer and a strategy for scaling up. Improvement has led to high accuracy in datasets with many small objects.

**Figure 2:**
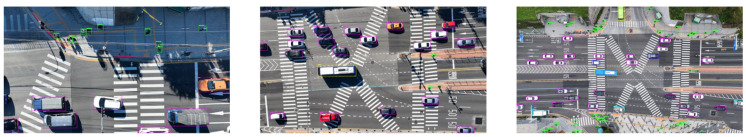
Examples of object detection using the PP-YOLOE+SOD algorithm.

Figure 2 shows examples of object detection by using the PP-YOLOE+SOD algorithm on drone footage captured at different altitudes (25 m, 50 m, 75 m).

To select an algorithm from the two candidates, metrics such as recall and precision were calculated from the confusion matrix. Table 6 presents the confusion matrix used to evaluate the performance of the models.

Precision refers to the proportion of correct predictions among all predictions made by the model. A high precision indicates that a large proportion of predictions are correct, although this metric does not consider FNs that the model could not predict.

$$Precision=\frac{True\;Positive}{True\;Positive+False\;Positive}$$

In contrast, recall indicates how well the model predicts the actual correct data. A high recall means that the model successfully captures the actual correct data with very few errors.

$$Recall=\frac{True\;Positive}{True\;Positive+False\;Negative}$$

The objective of this study is to establish the evaluation criteria for the creation of AI training data using existing algorithms. Data completeness is crucial when creating data for AI model training. This means that situations in which correct data are excluded from labeling should not occur. Therefore, recall was selected as the metric for comparing the model performance in the final algorithm selection. The recall performances when the two algorithms are applied to the dataset in this study are summarized according to the object type and filming altitude in Table 7 and Table 8.

Upon applying both algorithms to the object detection dataset obtained in this study, the PP-YOLOE+SOD algorithm achieved a slightly better recall of 0.9323 compared to 0.9318 for the PP-YOLOE+ algorithm. Therefore, the PP-YOLOE+SOD algorithm was selected as the object detection algorithm for generating the AI training data.

### 2.4. Selection of Performance Comparison Metrics for Object Detection Results

Recall, which ensures that as few correct predictions as possible are omitted from the actual correct data, was chosen as the evaluation factor to compare the performances of the two algorithms and select the optimal algorithm. An essential element that must be defined to derive the metrics for evaluating model performance, such as recall and precision, is the confidence threshold (CT). Each object detected by the object detection model has a confidence score (CS), which indicates the probability that the detected object belongs to one of the object categories defined in Table 7. The CT is a reference value that determines whether the detected object is considered a correct prediction. Only objects whose CS exceeds the CT are recognized as predictions during object detection. Setting a high CT can keep the number of FPs low but may result in many FNs. Conversely, a low CT can keep the number of FNs low and maintain high TP but can result in many FPs.

**Figure 3:**
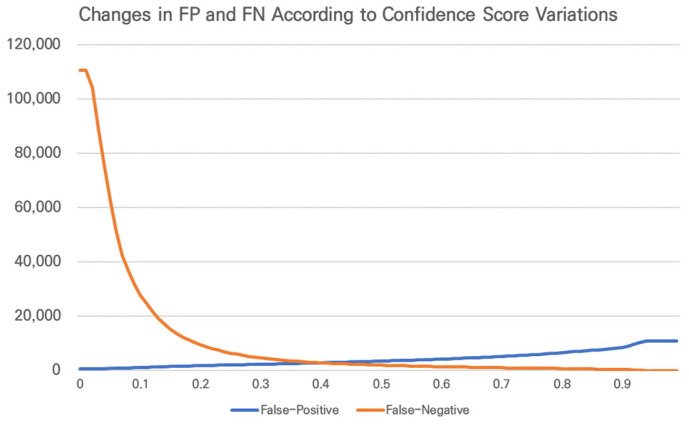
Changes in FP and FN as a function of changes in CT based on the results of object detection results from drone footage.

In the previous derivation of the recall, the CT was set to zero. It enables the detection of many TPs but may also result in excessive detection of FPs. This means that in the process of detecting complete objects, the model may detect non-actual objects as actual ones. Setting the CT to zero can achieve the ultimate goal of correctly detecting actual objects using the model. However, the downside is the occurrence of many FPs that are not correct but are detected as correct; therefore, a process to remove the FPs is required before the data can be used as AI training data. Therefore, by appropriately setting the CT to keep the FPs and FNs at a reasonable level, the automatically labeled data require minimal modifications, making it a necessary process for creating AI training data. Figure 3 illustrates the changes in the FPs and FNs as a function of the changes in the CS based on the data used in this study.

Different techniques are used to appropriately adjust the CT by considering the changes in the FNs and FPs. The receiver operating characteristic (ROC) curve and precision–recall (PR) curve are commonly used together with the F-beta score as a metric for model evaluation. The F-beta score is used to compensate for the situations where FP is not considered when choosing precision as the evaluation metric and where FN is not considered when choosing recall. The formula for calculating the F-beta score is as follows:

(1) $$F_{\beta }-Score=\left(1+\beta^{2}\right)\frac{Precision\times Recall}{\beta^{2}\times Precision+Recall}.$$

The F-beta score is a performance verification metric that considers both recall and precision. If beta is greater than one, recall is weighted more heavily; if it is less than one, precision is weighted more heavily. A beta value of one implies equal weighting. The weight is determined based on whether FN, which is not considered in evaluations using recall, or FP, which is not considered in evaluations using precision, is more critical. The value of beta can be adjusted depending on the data composition and model objectives. In the object detection domain, the beta value in the F-beta score for evaluating model performance is 0.5, 1, or 2 [48,49,50,51,52]. Therefore, the CT is adjusted, and the F_0.5_ score, F_1_ score, and F_2_ score are calculated for each scenario. Depending on the filming altitude and the type of object detected, the CT with the highest F-beta score is used as the basis for comparing the precision and recall. The result can be used to propose the criteria for determining the optimal CT for building the AI training data. The F_0.5_-socre is used in systems in which it is critical to minimize FP, such as spam filtering and advertisement recommendation systems. The F_1_ score is widely applied when a balance between FN and FP is needed. The F_2_ score is used in systems in which it is crucial to minimize FN, such as in the medical field, disaster response, and security systems. Since each metric has distinct characteristics, selecting the appropriate metric based on the situation is a crucial factor in evaluating the performance of the model.

## 3. Results and Discussion

### 3.1. Object Detection Performance Evaluation Based on the F-Beta Score and the CT Setting

As previously mentioned, setting the CT is necessary to adjust the FN and FP appropriately for the generation of AI training data. Therefore, experiments were conducted to adjust the CTs for each F-beta score and to determine whether each CT was appropriate for generating the AI training data. By varying the CT according to the type of target object, the precision and recall were calculated to derive the F-beta score. For each beta, the CT yielding the highest score was extracted. The ground truth (GT), TP, FN, FP, precision, and recall values derived from manual labeling for each CT are listed in Table 9.

The F-beta score was derived based on the changes in the CT, and the CT was set based on the highest F-beta score. The results show that the CT increased in the order F_0.5_, F_1_, F_2_, regardless of object type. The CT of the F_0.5_ score is generally almost twice as high as the CT of the F_2_ score, which is also the same as before. However, compared to the CTs of cars, trucks, and buses, the CTs of pedestrians and two-wheelers were lower, which means that the system is better at detecting relatively large objects despite the application of strict criteria. Specifically, two-wheelers had a lower Max score and CT than other objects, suggesting that it is an object that is difficult to detect using the object detection model. This was likely because a higher CT increases precision while recall decreases. In addition, the maximum F-beta score was similar for all object types, but it was slightly higher for larger objects such as cars, trucks, and buses than for smaller objects such as pedestrians and two-wheelers. This suggests that the object detection models tend to detect larger objects more effectively than smaller ones.

### 3.2. Establishing F-Beta Score Criteria for Object Detection Performance Evaluation Considering Error Correction Tasks

When creating the AI training data for AI model development, it is crucial to avoid problems with the completeness of the data, such as false detections or missed detections. Such issues result in problems that lead to inaccurate data, which can degrade the overall performance of the model or reduce its reliability [53,54]. Therefore, it is necessary to improve the completeness of the training data by adding missed FNs and removing falsely detected FPs based on the results derived from the object detection model. From this perspective, the F-beta score suitable for determining the CT should be determined, entailing tasks required to correct the FPs and FNs.

Correcting the FNs and FPs is an essential task in building AI training data, and this process inevitably requires human intervention. FN correction involves searching for undetected objects, creating bounding boxes, adjusting them to fit the actual objects, setting the object attributes, and saving the changes. In contrast, FP correction is simpler. It involves finding and deleting the bounding boxes that were mistakenly created for non-objects. Previous studies have shown that the correction of FPs takes significantly less time than that required for correcting the FNs [55]. Owing to the importance and difficulty of correcting FNs, models are being developed specifically to improve FN detection [56,57]. A simple comparison of the tasks involved in correcting the FNs and FPs during the generation of AI training data is listed in Table 10.

As the search for undetected objects in FN correction requires checking the entire video, whereas only the results of the object detection need to be checked in FP correction, FN correction will probably take more time. Even disregarding this task, adding an FN requires six mouse operations to create a bounding box, whereas deleting an FP requires only one mouse operation to select the corresponding bounding box. This means that adding an FN requires six times as much effort as deleting an FP. Therefore, to facilitate a quantitative comparison of the effort required to correct the FNs and FPs based on the F-beta score, the required effort was quantified using a 6:1 weighting, as indicated in Table 11.

Regardless of the object type, it was found that the quantified effort for error correction decreases in the order F_2_, F_1_, F_0.5_. The results show that F_0.5_ requires 135% of the effort for F_2_ and 107% of the effort for F_1_. These results reflect only the mouse operations associated with creating or deleting the bounding boxes during FN and FP corrections. The difference in effort is likely even greater when considering additional tasks such as finding missing objects (FN), selecting object attributes, and saving the results. Because the F_2_ score is commonly used in medical fields, such as in COVID-19 detection and tumor detection, the reduction of FNs is crucial; it is considered a crucial metric for building AI training data, although FN correction requires more effort [58,59]. Using the F_2_ score to assess the labeling performance of models offers the advantage of optimization by object type compared to mAP. When evaluating performance on identical videos, the mAP yields the overall efficacy of the model. Conversely, the F_2_ score, similar to average precision (AP), evaluates performance based on specific object categories or overall and has the difference that the CT is optimized for each object individually. Although AP and mAP are effective tools for evaluating model performance by considering both precision and recall, the F-beta score could be more effective for AI dataset construction, where it is necessary to weigh the correction of FP and FN. While AP and mAP can represent the overall performance of the model, the F-beta score is more suitable for deriving and applying the optimal CT through model adjustments. It can also be observed that F_2_ is significantly different from F_1_ and F_0.5_ when classified by object type. In the case of the bus with the fewest objects, the quantified effort of F_2_ and F_0.5_ is approximately twice as high, whereas it is 1.4 times as high for pedestrians who have the most objects. This indicates that the quantified effort is not biased due to the large number of objects but rather represents a similar trend for all objects. It can be concluded that despite the imbalance of the existing data, the performance of building semi-automated AI training data can be reviewed using F_2_.

## 4. Conclusions

This study evaluated the model performance and established the evaluation criteria for optimal object detection performance to create training data for training new AI models by applying existing object detection models to drone-captured urban traffic-environment videos and using manual correction processes. The F-beta score was set as an evaluation criterion to determine the CT by finding a balance between FNs and FPs when optimizing the object detection results. By adjusting the beta value of the F-beta score to 0.5, 1, and 2, the CTs were derived based on the object type, and the object detection results were compared based on the CT settings. It was confirmed that the CT decreased with increasing beta, and reducing the FNs increased the FP. To construct AI training data using object detection results from conventional object detection models, a modification process is required to ensure the completeness of the data. Therefore, the F-beta score for evaluating the object detection performance was determined based on actual error correction tasks. As a result of analyzing the task to modify FPs and FNs, it was found that adding an FN requires at least six times more effort than deleting an FP. Therefore, FNs and FPs were evaluated with a 6:1 weighting according to the beta used. The determination of CTs using F_0.5_ required 135% more effort than those with F_2_, whereas using F_1_ required 107% more effort than those with F_2_. This indicates that using the F_2_ score for CT determination and object detection is more efficient in terms of building AI training data. Actual footage data were used, and previously published algorithms were applied to validate the F_2_ score as an evaluation metric for the construction of semi-automated AI training datasets. In the future, the results of object detection with different algorithms on diverse datasets will be analyzed using the F_2_ score. Comparison and analysis with other existing metrics will provide a clearer picture of the utility of the metrics.

## Figures and Tables

**Table 1:** Details of the video recordings.

	Filming Location	Schedule	Drone Type
1	Seodaemun-gu, Seoul	8 November 2022	DJI Mavic 3
2	Ilsanseo-gu, Goyang-si	13 October 2023 16 October 2023	Argosdyne AQUILIA-2

**Table 2:** Classification of moving objects for object detection.

No.	1	2	3	4	5
Class	Pedestrian	Two-wheeler	Car	Truck	Bus

**Table 3:** Resolution, number of videos, and total number of objects in the footage.

Altitude	Resolution	Number of Videos	Total Number of Objects
25 m	3840 × 2160	28	Total of 7670 objects. Average of 47.06 objects per video
50 m		65	
75 m		70	
100 m	5472 × 3080	117	Total of 3303 objects. Average of 28.2 objects per video

**Table 4:** Criteria for the selection of object detection algorithms.

Criterion	Description
Mission type	Algorithms for object detection (Object Detection) with bounding box labeling.
Target data	Algorithms that are evaluated using drone-captured video data.
Object category	Algorithms that are evaluated for detecting moving objects, such as pedestrians, vehicles, and personal mobility (motorcycles, bicycles).
Algorithm accuracy	Algorithms verified using benchmark datasets in similar fields.
Algorithm usability	Algorithms with open-source codes that have been shared and improved by many users.

**Table 5:** Comparison between candidate algorithms.

Algorithm	Mission Type	Target Data	Object Category	Algorithm Accuracy		Algorithm Usability (Git Stars)
				Benchmark	mAP0.5	
PP-YOLOE+ (largesize-L)	Object detection	Drone video (VisDrone)	10 types, including pedestrians, cars, bicycles	VisDrone (val)	66.7	11.2 k
				VisDrone (test)	54.7	
PP-YOLOE+ SOD (largesize-L)	Object detection	Drone video (VisDrone)	10 types, including pedestrians, cars, bicycles	VisDrone (val)	65.9	11.2 k
				VisDrone (test)	55.1	

**Table 6:** Confusion matrix.

		Real	
		True	False
Prediction	True	True positive (TP)	False positive (FP)
	False	False negative (FN)	True negative (TN)

**Table 7:** Performance of PP-YOLOE+ on the dataset in this study.

Object Type	Filming Altitude				Overall
	25	50	75	100	
Pedestrian	1.0000 (63/63)	0.9628 (596/619)	0.9272 (2688/2899)	0.8207 (563/686)	0.9163 (3910/4267)
Two-wheeler	1.0000 (12/12)	0.8713 (440/505)	0.7571 (589/778)	0.8275 (307/371)	0.8091 (1347/1666)
Car	1.0000 (78/78)	0.9970 (673/675)	0.9683 (1465/1513)	0.9964 (1921/1928)	0.9864 (4137/4194)
Truck	1.0000 (13/13)	0.9063 (87/96)	0.9948 (192/193)	0.9914 (230/232)	0.9775 (522/534)
Bus	1.0000 (12/12)	1.0000 (56/56)	0.9873 (156/158)	0.9767 (84/86)	0.9872 (308/312)
Overall performance	1.0000 (178/178)	0.9493 (1852/1951)	0.9186 (5090/5541)	0.9401 (3105/3303)	0.9318 (10,225/10,973)

**Table 8:** Performance of PP-YOLOE+SOD on the dataset in this study.

Object Type	Filming Altitude				Overall
	25	50	75	100	
Pedestrian	1.0000 (63/63)	0.9677 (599/619)	0.9262 (2685/2899)	0.8032 (551/686)	0.9135 (3898/4267)
Two-wheeler	1.0000 (12/12)	0.8733 (441/505)	0.7776 (605/778)	0.8140 (302/371)	0.8163 (1360/1666)
Car	1.0000 (78/78)	1.0000 (675/675)	0.9650 (1460/1513)	0.9979 (1924/1928)	0.9864 (4137/4194)
Truck	1.0000 (13/13)	0.9479 (91/96)	0.9948 (192/193)	0.9957 (231/232)	0.9869 (527/534)
Bus	1.0000 (12/12)	1.0000 (56/56)	0.9873 (156/158)	0.9767 (84/86)	0.9872 (308/312)
Overall performance	1.0000 (178/178)	0.9544 (1862/1951)	0.9201 (5098/5541)	0.9361 (3092/3303)	0.9323 (10,230/10,973)

**Table 9:** Results of CT settings by object type and beta level for the dataset in this study.

Category	F-Beta Score		CT (in Max Score of Beta)	GT	TP	FN	FP	Precision	Recall
	Type	Max Score							
Pedestrian	F_0.5_	0.79	0.5	4267	2787	1480	557	0.83	0.65
	F_1_	0.75	0.39	4267	3170	1097	965	0.77	0.74
	F_2_	0.77	0.27	4267	3490	777	1995	0.64	0.82
Two-wheeler	F_0.5_	0.53	0.4	1666	603	1063	396	0.60	0.36
	F_1_	0.48	0.35	1666	702	964	586	0.55	0.42
	F_2_	0.48	0.26	1666	862	804	1499	0.37	0.52
Car	F_0.5_	0.81	0.83	4194	3031	1163	608	0.83	0.72
	F_1_	0.81	0.62	4194	3491	703	981	0.78	0.83
	F_2_	0.83	0.44	4194	3610	584	1372	0.72	0.86
Truck	F_0.5_	0.91	0.59	534	381	153	11	0.97	0.71
	F_1_	0.87	0.36	534	456	78	59	0.89	0.85
	F_2_	0.88	0.24	534	488	46	135	0.78	0.91
Bus	F_0.5_	0.94	0.73	312	255	57	5	0.98	0.82
	F_1_	0.92	0.52	312	279	33	17	0.94	0.89
	F_2_	0.92	0.36	312	288	24	30	0.91	0.92

**Table 10:** Tasks involved in the correction of FNs and FPs.

Error Type	Correction Task
FN	➀ Review the entire video to find undetected objects. ➁ Select two points to create a bounding box. ➂ Adjust the size of the bounding box by modifying the four points. ➃ Select the object type. ➄ Save.
FP	➀ Search for the incorrectly detected object. ➁ Select the bounding box. ➂ Delete.

**Table 11:** Quantification of effort required for FN and FP corrections.

F_beta_ Score	Category	FN	FP	Quantified Effort
F_0.5_	Pedestrian	1480	557	9437
	Two-wheeler	1063	396	6774
	Car	1163	608	7586
	Truck	153	11	929
	Bus	57	5	347
	**Overall**	**3916**	**1577**	**25,073**
F_1_	Pedestrian	1097	965	7547
	Two-wheeler	964	586	6370
	Car	703	981	5199
	Truck	78	59	527
	Bus	33	17	215
	**Overall**	**2875**	**2608**	**19,858**
F_2_	Pedestrian	777	1995	6657
	Two-wheeler	804	1499	6323
	Car	584	1372	4876
	Truck	46	135	411
	Bus	24	30	174
	**Overall**	**2235**	**5031**	**18,441**

## Data Availability

Data are contained within the article.
